# Rationale and design of the Adapted Physical Activity in advanced Pancreatic Cancer patients (APACaP) GERCOR (Groupe Coopérateur Multidisciplinaire en Oncologie) trial: study protocol for a randomized controlled trial

**DOI:** 10.1186/s13063-015-0983-8

**Published:** 2015-10-12

**Authors:** Cindy Neuzillet, Mathieu Vergnault, Franck Bonnetain, Pascal Hammel

**Affiliations:** Digestive Oncology Department and UMR1149, Beaujon University Hospital, Hôpitaux Universitaires Paris Nord Val de Seine (HUPNVS), Assistance Publique-Hôpitaux de Paris (AP-HP), 92110 Clichy La Garenne, France; Visio Activités Sportives Interactives (V@si SARL), 34270 Saint Mathieu de Tréviers, France; Methodology and Quality of Life in Oncology unit (EA 3181) and Quality of Life and Cancer Clinical Research Platform, Besançon University Hospital, 25000 Besançon, France

**Keywords:** Fatigue, Health-related quality of life, Insulin resistance, Locally advanced, Metastatic, Pancreatic adenocarcinoma, Physical exercise, Study protocol, Supportive care, Survival

## Abstract

**Background:**

Exercise during chemotherapy is a promising strategy to reduce fatigue and improve health-related quality of life (HRQoL). It has been shown to be feasible and efficient in various cancers, including advanced-stage cancers. Effects of physical activity have never been explored in advanced pancreatic ductal adenocarcinoma (PDAC). We aim to evaluate the effects of an exercise intervention in this setting.

**Methods:**

This randomized, national, multicenter, interventional study will examine the effectiveness of an unsupervised, home-based, 16-week adapted physical activity (APA) program. Specificities of advanced PDAC for the implementation of the APA program will be taken into account (healthy volunteer as physical activity partner instead of patient groups, nutritional management). The main inclusion criteria are: patients with histologically confirmed, unresectable PDAC; scheduled for chemotherapy; performance status 0–2; age ≥ 18; and physical activity partner. In total, 200 patients will be randomized into either the APA program (aerobic and resistance exercises) in addition to usual care (including chemotherapy at the investigator’s choice), or usual care. The primary objective will be the effect on fatigue (Multidimensional Fatigue Inventory, MFI-20) and HRQoL (European Organization for Research and Treatment of Cancer-Quality of Life-C30 questionnaire, EORTC-QLQ-C30; co-primary endpoint) at week 16. As secondary objectives, the effects of the exercise intervention on pain, anxiety, depression, nutritional status, insulin resistance, tolerance of chemotherapy, survival, and adherence to the APA program will be evaluated.

**Discussion:**

Patients with advanced PDAC are strongly affected by fatigue, and are thus likely to benefit from an exercise intervention. Moreover, exercise may have a potential beneficial effect on tumor outcome by reducing insulin resistance and insulin/insulin-like growth factor-1 (IGF-1) secretion. However, an exercise intervention may appear challenging due to multiple PDAC-related symptoms such as fatigue, depression, pain, and denutrition. We hypothesize that an APA program taking into account specific characteristics of PDAC may improve symptoms and HRQoL. If demonstrated to be feasible and effective, such APA programs will be systematically proposed to patients with advanced PDAC in addition to usual care.

**Trial registration:**

ClinicalTrial.gov Registration number: NCT02184663; Registration date: 2 July 2014.

**Electronic supplementary material:**

The online version of this article (doi:10.1186/s13063-015-0983-8) contains supplementary material, which is available to authorized users.

## Background

### Current knowledge about physical activity in cancer patients

Physical activity is associated with a decreased risk of cancer, including colorectal, breast, and endometrial cancers [1–4]. According to the World Health Organization (WHO), 30 minutes of physical activity five times per week may reduce the risk of developing colorectal or breast cancer by about 25 %.

There is accumulating evidence that physical activity is also beneficial for patients with cancer in reducing disease and/or treatment-induced symptoms (including pain, fatigue, and anxiety/depression), and improving health-related quality of life (HRQoL) as well as treatment tolerance and adherence [5–8]. Fatigue in cancer patients is multifactorial and can be related to the disease itself, treatment, and bed rest, leading to deconditioning. Deconditioning, that is, loss of physical (cardiorespiratory function and muscle strength) and psychological fitness caused by reduced physical activity, is one of the main causes of cancer-related fatigue [9]. Paradoxically, rest may have deleterious effects on these patients, while physical exercise is the best way to reduce deconditioning and fatigue [10–12]. Several studies have reported a 30 % reduction in cancer-related fatigue and maintained cardiorespiratory fitness and muscle strength with higher physical activity, even in advanced-stage cancer [8].

A beneficial effect of physical activity on overall survival (OS) has also been reported in the most frequent cancers such as those of the breast, colon, and prostate [13]. Exercise reduces circulating estrogen and sex hormone binding globulin (SHBG) levels in sex hormone-dependent cancers, improves insulin profile (insulin-related pathways including insulin-like growth factor 1 [IGF-1]), reduces inflammation, and increases natural killer and T-cell-mediated immunity [13–18]. Moreover, physical activity may improve survival through its impact on HRQoL, a prognostic indicator of survival in various cancers including pancreatic cancer [19, 20].

The French National Cancer Institute (INCa) (http://www.afsos.fr/) promotes physical activity in the prevention and management of cancer at any time. The French association for supportive care in cancer (Association Francophone pour les Soins Oncologiques de Support, AFSOS, http://www.afsos.fr/), the American College of Sports Medicine (ACSM), and the American Cancer Society (ACS) have edited guidelines for “adapted physical activity” (APA) in cancer patients based on published data [21–24]. In France, but also in many other countries, adapted physical activity (APA) is a concept defined by the International Federation of Adapted Physical Activity (IFAPA) as “*a cross-disciplinary body of practical and theoretical knowledge directed toward impairments, activity limitations, and participation restrictions in physical activity. It is a service delivery profession and an academic field of study that supports an attitude of acceptance of individual differences, advocates access to active lifestyles and sport, and promotes innovative and cooperative service delivery, supports, and empowerment. Adapted physical activity includes, but is not limited to, physical education, sport, recreation, dance, creative arts, nutrition, medicine, and rehabilitation.*” [25]. In the cancer setting, the term APA is used for exercise interventions involving professional trainers who have graduated with an academic degree in APA. Implementation of an APA program in patients with cancer implies a multidisciplinary collaboration between the cancer-care medical team and APA professionals. The APA program should be individualized according to the patient (physical fitness, exercise type preferences, psychological functions, and expectations), the cancer (stage, treatments, and tolerance), and the social environment. A combined aerobic exercise and resistance-training program is hypothesized to be the most efficient way to improve physical fitness and decrease fatigue and therefore should be favored [21–24]. Patient adherence to the APA program is crucial for its efficacy. Performing exercise in groups of patients having similar physical capabilities and under the supervision of an APA professional whenever possible is the best way to ensure patient motivation. The APA program also requires the contribution of nutrition specialists (dietitians or physicians) to balance energy intake with energy expenditure. Little is known about the optimal duration of exercise interventions, and no specific recommendation from evidence-based guidelines exists [24]. In the Cochrane meta-analysis review of randomized controlled trials evaluating the effectiveness of exercise interventions on health-related quality of life (HRQoL) in people with cancer during active treatment [7], the length of exercise intervention varied greatly between trials (3–26 weeks), with a modal duration of 12 weeks. Similarly, a modal duration of exercise interventions of 12 weeks was reported in the Cochrane meta-analysis of trials that investigated the effect of exercise on the management of cancer-related fatigue [8]. In patients who exercised during treatment, improvements in global health and physical and emotional functioning compared to baseline values were found at week 12 [24]. Noticeably, in these studies, patients were allowed to continue exercise after the end of the intervention period.

### Epidemiology and management of pancreatic ductal adenocarcinoma (PDAC)

Pancreatic ductal adenocarcinoma (PDAC) is the second cancer of the digestive tract in incidence and one of the poorest prognostic tumors with a 5-year survival rate of less than 5 % [26]. More than 80 % of patients have an advanced, unresectable disease at diagnosis. Therapeutic options remain limited in this setting [27]. Treatment consists mainly of chemotherapy with weekly (gemcitabine or gemcitabine plus *nab*-paclitaxel regimens) or bi-weekly regimens (fixed-dose rate [FDR] gemcitabine, the combinations of 5-fluorouracil, irinotecan, and oxaliplatin [FOLFIRINOX], or gemcitabine and oxaliplatin [GEMOX]). Radiation therapy is limited to selected patients with non-metastatic disease. To date, there are no data about the role of APA in patients treated for an advanced PDAC.

### Hypothesis for beneficial effects of exercise intervention in patients with advanced PDAC

Fatigue is a common disease-related symptom in patients with advanced PDAC that contributes to impaired HRQoL [28, 29]. We hypothesize that physical exercise may limit physical deconditioning and reduce fatigue in these patients. Physical activity may have a beneficial effect on pain, anxiety, and depression, symptoms affecting more than half of PDAC patients, and may result in a global improvement of HRQoL and in delayed deterioration of HRQoL [30]. Moreover, an improvement in HRQoL may translate into a survival benefit [20].

There is consistent evidence on the relationship between insulin resistance, insulin and IGF-1 secretions, and pancreatic carcinogenesis [31–37]:

- Obesity and type II diabetes (metabolic syndrome) are risk factors for PDAC (relative risk [RR] = 1.2–1.6 and 1.8–2.0, respectively) and physical activity may have a protective effect (RR = 0.72),
- Metformin, a drug that decreases insulin resistance, reduces the risk of developing PDAC in *in vivo* preclinical studies and in observational clinical studies of patients with type II diabetes,
- Insulin receptor is overexpressed in PDAC, and insulin and IGF-1 stimulate proliferation of PDAC cells *in vitro*,
- Some polymorphisms of IGF, IGF receptor, and IGF-binding protein are associated with an increased risk of PDAC and shorter survival in PDAC patients,
- Weight loss, impaired glucose tolerance and diabetes, along with peripheral insulin resistance are frequently observed in PDAC patients.

Physical exercise has been associated with reduced insulin resistance and insulin and IGF-1 secretions [13]. In addition, PDAC usually causes an intense inflammatory reaction that contributes to tumor-related cachexia [38]. Exercise can modulate this process and may have a beneficial effect on disease outcome (progression-free survival [PFS] and OS) in patients with advanced PDAC.

### Specificities of advanced PDAC affecting the implementation of the APA program

Sessions in groups are suitable to maintain patient motivation, particularly in an adjuvant setting (for example, for breast and colon cancer). In women with breast cancer, being part of a supportive social environment is acknowledged in motivational and behavioral theories as a key factor to health behavior change and stands among the strongest incentives for exercise motivation [39]. However, we believe that patients with advanced PDAC are not suitable for group sessions. These patients have a poor prognosis and can deteriorate during the exercise intervention period. Therefore, regular meetings of patients treated for PDAC may lead to negative and counterproductive psychological impact on the group. For this reason, we propose to include a “physical activity partner,” a healthy volunteer, chosen by the patient, to attend the exercise sessions. This practice is expected to sustain the patient motivation and to be beneficial for the partner in terms of psychological outcome, while avoiding the potentially deleterious psychological effects of the patient group [40]. Such organization will imply a psychological evaluation of the partner to verify that he/she is not experiencing psychological burnout.

Patients with PDAC often suffer from weight loss due to an inadequate dietary intake combined with increased energy expenditure [41]. Denutrition is experienced by 70–80 % of PDAC patients and is multifactorial in origin including inflammatory and hypercatabolic syndrome, stenosis of the digestive tract, cholestasis, exocrine pancreatic insufficiency, diabetes, anxiety, depression, and chemotherapy adverse effects (nausea/vomiting, mucitis, diarrhea, and loss of appetite). Patients with PDAC appeared to have a significantly increased basal resting energy expenditure (REE) and spontaneously reduced physical activity level compared with healthy individuals [41]. As the APA program is expected to increase energy expenditures, nutritional management will be crucial for monitoring and adapting food intakes to ensure that patients meet their nutritional needs.

There is consistent evidence that a 12-week intervention is the minimal duration of exercise intervention to improve HRQoL and fatigue in patients with cancer [7, 8, 24]. In the setting of pancreatic ductal adenocarcinoma (PDAC), median progression-free survival (PFS) in patients with metastatic disease ranges from 3 to 4 months with gemcitabine, from 5 to 6 months with gemcitabine plus *nab*-paclitaxel or the FOLFIRINOX combination regimens, and from 9 to 10 months in patients with locally advanced PDAC [42–44]. Deterioration of HRQoL is closely related to disease progression and survival, and can be affected by a change in chemotherapy regimen in the case of tumor progression [20]. Thus, evaluation of HRQoL can be biased in many ways by disease progression. Therefore, we propose a 16-week intervention and HRQoL and fatigue data evaluation at week 16, since most patients are expected to remain free of disease progression during this time period and to remain on the same chemotherapy regimen. In addition, evaluation of tumor status under treatment in advanced PDAC is usually performed every 8 weeks; thus, this length of exercise intervention was judged as well suited for the timing of tumor evaluation.

## Methods/design

### Study objectives and hypotheses

The APACaP (*ActivitéPhysiqueAdaptée et chez des patients ayant unCancer duPancréas non résécable*; adapted physical activity in patients with unresectable pancreatic adenocarcinoma) study aims to assess the efficacy of an unsupervised 16-week APA program on physical fatigue (Multidimensional Fatigue Inventory, MFI-20) and targeted dimensions of HRQoL (global QoL, fatigue, physical functioning, and pain; European Organization for Research and Treatment of Cancer-Quality of Life-C30 questionnaire, EORTC-QLQ-C30), unified as the co-primary endpoint (single sufficient design) at the end of the intervention in advanced PDAC patients. Secondary objectives include assessing the beneficial effects on pain, other HRQoL dimensions, depression, nutritional status, insulin resistance, cancer-treatment tolerance, PFS, and OS.

### Design

**Fig. 1:**
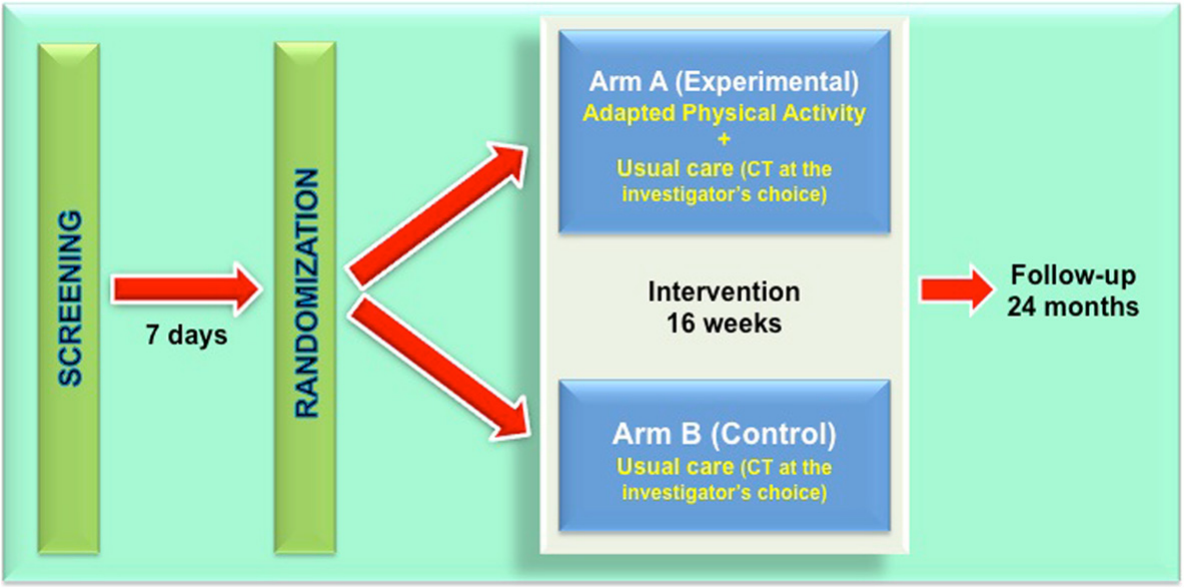
The APACaP study design. CT: chemotherapy

The APACaP study is designed as a national, prospective, multicenter, randomized, controlled trial to test the efficacy of an APA program, in which patients will be allocated either to the intervention group invited to perform exercise in addition to usual care, or to the usual care control group (Fig. 1). Because blinding of participants toward allocation is not feasible, this study will have an open-label design.

### Patients

Patients with histologically confirmed, previously untreated, unresectable (locally advanced or metastatic) PDAC who meet the following criteria will be eligible for the APACaP study: scheduled for chemotherapy; Eastern Cooperative Oncology Group (ECOG) performance status (PS) ≤ 2 (Additional file 1: Table S1); age ≥ 18 years; at least one bi-dimensionally measurable lesion according to the Response Evaluation Criteria In Solid Tumors (RECIST) v1.1 criteria [45]; designated physical activity partner; signed and dated informed consent; and registration in a national health care system (CMU included). Patients will be excluded if their cardiovascular, respiratory, psychiatric, musculoskeletal, or neurological condition contraindicates exercise practice. Participants of other clinical studies will be eligible for the APACaP study if the primary endpoints of both studies do not overlap.

### Ethics and regulatory considerations

Written informed consent will be obtained from all patients. The current study is to be conducted in accordance with globally accepted standards of Good Clinical Practice (ICH-E6), European Directive 2001/20/EC, and the revised version of the Declaration of Helsinki set out in the European Directive, as well as with the Code de Santé Publique specific to France. The protocol has been submitted for formal approval of the study conduct to ensure that the study meets the local regulations to the Health Authorities, that is, the Agence Nationale de Sécurité du Médicament et des produits de santé (ANSM), and to a properly constituted Ethics Committee. The study was approved by Health Authorities (ANSM), and full ethical approval was obtained from a unique Ethics Committee for all centers involved (CPP Ile-de-France VI N 32–14; ID RCB: 2014-A00228-39), in compliance with French regulations. The study has been registered on ClinicalTrial.gov (NCT).

### Recruitment and randomization

This study is promoted by the Groupe Coopérateur Multidisciplinaire en Oncologie (GERCOR) and will be conducted in 16 centers in France (managed by the GERCOR and Partenariat de Recherche en Oncologie DIGEstive [PRODIGE] cooperative groups). Newly diagnosed patients with advanced PDAC identified as eligible will be informed about the program and invited to participate in the study during a regular outpatient visit.

The clinician will assess the patient eligibility and will provide each patient and/or a legal representative with relevant, comprehensive, verbal and written information regarding the objectives and procedures of the study as well as their possible risks. The patient will be given a 1-week reflection period (up until the first chemotherapy cycle) in order to inquire about study details. All questions raised by the patient should be answered in a satisfying manner. The investigator will inform each patient about his/her right to withdraw from the study at any time for any reason. A signed informed consent will be obtained from each patient and/or legal representative prior to undertaking any study-related procedure.

Eligible patients will be randomized in a 1:1 ratio by a central computer-assisted procedure centralized at the GERCOR data center. Randomization will be performed using a minimization technique and will be stratified per center, cancer stage (locally advanced versus metastatic), chemotherapy schedule (weekly versus biweekly regimen), ECOG PS (0–1 versus 2), and baseline physical activity level (low, moderate, or intense according to the Global Physical Activity Questionnaire [GPAQ]). Each patient who enters into the study will receive a unique subject identification number.

### Study protocol

#### Control group

Participants assigned to the control group will receive usual care (that is, with no physical activity intervention) including chemotherapy at the investigators’ choice (according to the patient general condition and biology, comorbidity, and tumor stage: weekly gemcitabine or gemcitabine plus *nab*-paclitaxel regimens; biweekly FDR gemcitabine, FOLFIRINOX, or GEMOX), outpatient clinical visits according to the regular schedule, nutritional support according to the national recommendations (Société Francophone de Nutrition Clinique et Métabolisme [SFNEP]), and tumor evaluation based on carbohydrate antigen 19–9 (CA 19–9) serum levels and thoraco-abdominopelvic computed tomography (TAP-CT) every 8 weeks.

#### Intervention group

Participants assigned to the intervention group will receive usual care plus a 16-week APA program.

The APA program, designed according to the AFSOS recommendations, will be directed by an APA professional trainer selected from a list of certified professionals chosen for the study. Exercise sessions will be implemented in addition to daily life activities. The exercise training program will consist of one supervised demonstration session with the APA professional, and thereafter the training will be continued as unsupervised, home-based exercise training sessions.

Two types of exercises will be proposed: 1) aerobic exercises including walking, and/or Nordic walking, and/or cycling, according to patient preference and 2) resistance exercises using elastic bands to maintain or increase arm and leg muscle strength.

Duration, frequency, and intensity of exercises will be adapted for each patient by the APA professional according to standardized guidelines. Patient fitness before treatment (estimated by the GPAQ), deconditioning level, and baseline physical evaluation (6-minute walking test and maximal strength test) will be considered. The APA professional will define exercise intensity based on baseline patient physical evaluation. Aerobic exercises will be performed with an intensity that will allow the patient to speak comfortably. The muscle-strengthening program will consist of repetitions performed at 50 % of baseline maximal strength test. One-repetition maximum (1-RM) is a measure of muscular strength; it is the highest weight that can be lifted for one repetition and one repetition only. Elastic bands (TheraBands®), available as the eight color-coded resistance level bands, are frequently used to assess the 1-RM [46, 47]. The APA professional will measure the distance from starting position to ending position during exercise with the elastic band to determine its deformation. After a warm-up without the elastic band and based on the APA professional assumption of the patient’s approximate strength, the appropriate band will be chosen for exercise. If the patient can perform more than ten repetitions with good form, a higher resistance band will be tested. The amount of deformation, the elastic band color, and the number of repetitions are required parameters to calculate a 1-RM. The 1-RM test is calculated based on the Brzycki equation: 1-RM = weight loaded/ (1,0278 - (0,0278 x number of repetitions)) [48]. The weight loaded is defined for each color of elastic bands depending on the level of deformation [49].

Physical activity sessions will comprise exercises for warming up followed by aerobic or resistance exercises and a recuperation period. The warming-up and recuperation periods will account for at least 30 % of the session duration. The aim of the APA program is to gradually reach a total of 30 minutes of aerobic training, three to five times per week (according to physical condition), and to perform strengthening activities at least two times per week for all the major muscle groups, excluding the warming-up and recuperation periods. A lower intensity activity week will be planned every 4 weeks to prevent patient exhaustion.

**Fig. 2:**
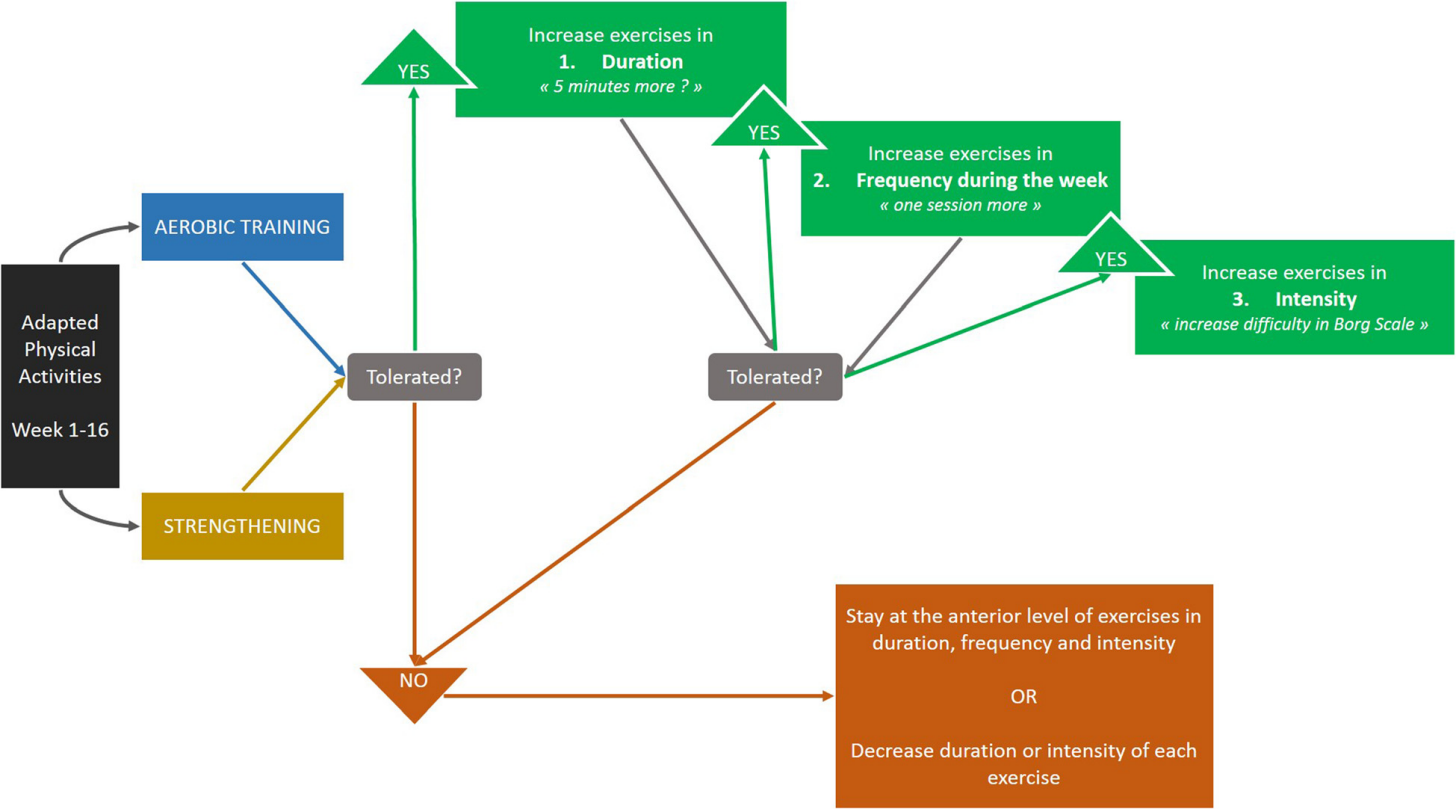
Mapping of the progression steps of the 16-week adapted physical activity (APA) program

Patients will be asked to report on their APA program in a dedicated activity booklet. Photographs and explanations for good positioning and proper execution of exercises will be provided to patients. The APA professional will monitor weekly the program tolerance and adherence during a video call (a webcam will be provided to the patient if necessary). The booklet can be used to help patients recall exercises performed and difficulties encountered. The video call will allow the APA professional to evaluate leisure activities, sessions of aerobic training, and strengthening exercises, and to assess the patient tolerance and difficulties in completing the physical activity level. Depending on this evaluation, the APA program will be adapted according to the scheme illustrated by Fig. 2. Difficulty of activities will be increased in duration, then in frequency, and ultimately in intensity. Exercise intensity will be enhanced only if the patient tolerates an increase in duration and frequency.

A supervised session with elastics can be performed during video call on patient demand.

After completion of the 16-week study program, the patient will be offered the option of pursuing the physical exercises.

The APA program will be temporary interrupted in the following cases: uncontrolled infection, thromboembolic event (first 7 days of anticoagulant therapy), uncontrolled pain, symptomatic anemia or hemoglobin level < 10 g/dl, platelet counts < 50,000/mm^3^, and a major surgery.

Termination of the patient participation in the study can occur in the following situations: on patient’s request, deterioration of general condition with ECOG PS > 2, uncontrolled symptoms, clinical problem definitively contraindicating exercise practice, clinicians’ or trainers’ decision in the best interest of the patient, serious adverse event (AE) resulting from physical exercise or preventing the patient from pursuing exercise, loss to follow-up, and major protocol deviation.

The patient will be asked to designate a physical activity partner (a healthy family member/relative or friend) who can attend (without obligation of performing exercises) and support the patient for at least one exercise session per week. The physical activity partner will be given all needed information and will be allowed to attend the patient visits with the physical activity trainer. Psychological follow-up of the physical activity partner will be scheduled using the Hospital Anxiety and Depression Scale (HADS).

The APA program will be associated with a nutritional intervention supervised by a dietitian. Nutritional management will be designed according to the SFNEP recommendations. Practically, patient nutritional status will be assessed based on clinical (actual weight, weight loss, and body mass index) and biological (albumin, prealbumin, and C-reactive protein [CRP]) parameters. The daily calorie and protein requirements will be calculated using the Harris-Benedict equation for patient basal energy expenditures. To determinate the total daily caloric requirements, two variables, the individual cancer-related stress factor (REE x 1.3) and the activity factor (REE x 1.4 a 1.6) will be considered. A three-day food intake evaluation will be performed. Nutritional intervention (oral feeding and enteral and/or parenteral nutrition) will be decided according to the SNFEP guidelines.

### Safety

All AEs reported by the patient or observed by the clinician and the APA professional (sports accidents, exhaustion, or injuries) will be recorded. All serious AEs will be reported to the ANSM and to the accredited European Commission that approved the study protocol.

It will be the responsibility of the clinician investigator to record all the relevant information regarding any event occurring during the APA program. The investigator will be requested to assess the relationship between the intervention and the occurrence of each AE using clinical criteria. Alternative causes such as natural history of the underlying diseases, concomitant therapy, other risk factors, and the temporal relationship of an event to the intervention will be considered and investigated.

The investigators and all other appropriate persons will be informed of findings that could adversely affect the safety of patients in a timely manner during the study duration.

### Study endpoints and measures

#### Primary outcome

The primary outcome of the APACaP study is to assess the effects of the APA program on fatigue (one targeted dimension) and HRQoL (four targeted dimensions), unified as a co-primary endpoint, at the end of a 16-week intervention in patients with advanced PDAC. We hypothesize that exercise intervention will significantly improve at least one of these five parameters without significantly deteriorating the others.

Fatigue will be measured using the validated French version of the MFI-20 questionnaire [50, 51]. The MFI-20 is a 20-item self-reported instrument, organized into five scales: general, physical, mental fatigue, and reduced activity and motivation, and is designed to assess multiple fatigue dimensions. The targeted dimension will be the physical fatigue scale. A change of two or more units will be considered as a clinically relevant change [52].

The EORTC-QLQ-C30 questionnaire comprising five functional scales (physical, role, emotional, cognitive, and social), one global QoL scale, and a 10-item symptom scale (including fatigue and pain) will be used to measure HRQoL [53]. The four targeted dimensions will be global QoL, fatigue, physical functioning, and pain. A change of five or more units will be considered as the minimal clinically important change [54].

#### Secondary outcomes

Secondary outcomes will consist in the longitudinal analysis of fatigue and HRQoL, and the effects on pain, depression, nutritional status, insulin resistance, cancer-treatment tolerance, and survival.

Longitudinal analysis of fatigue and HRQoL will be performed using a linear mixed-effects regression model (repeated measures analysis of variance [ANOVA]) to test randomization and time effects and a time-treatment interaction. In case of non-random missing data, pattern mixture models will be used. Time until definitive deterioration (TUDD) of targeted scores will be defined as the interval between the measurement of HRQoL at baseline (before randomization) and the date of occurrence of a 5-point or greater HRQoL score deterioration, no improvement thereafter, or death from all causes [20, 55, 56]. If deterioration is not observed, patients will be censored at the last date at which the patient has filled out the questionnaire.

Pain will be assessed using a visual analogue scale (VAS), analgesics consumption, and the French validated version of the Brief Pain Inventory-Short Form (BPI-SF) questionnaire [57, 58]. The BPI-SF questionnaire is a 15-item scale evaluating five parameters of pain: severity, impact on daily function, location, pain medications, and amount of pain relief in the past 24 hours or the past week. Two scores will be calculated to assess the severity of pain and the impact of pain on daily functions.

Anxiety and depression will be rated using the French validated version of the HADS questionnaire and based on anxiolytic and antidepressant consumption [59, 60]. The HADS questionnaire evaluates emotional state reported by the patient during the week before the assessment date. It consists of 14 items including seven for anxiety and seven for depression. Each item is rated on a 0–3 rating scale (3 = highest intensity level of feeling). The anxiety and depression scores will be calculated by the sum of the corresponding seven items. A score > 11 will be considered as significant for the presence of an anxiety or depression problem.

Nutritional status will be evaluated based on weight, body mass index (kg/m^2^), body composition (bioimpedancemetry method in equipped centers), food intake (assessed using a VAS), and biological parameters (albumin, prealbumin, and CRP).

Insulin resistance will be determined based on levels of fasting plasma glucose, insulin, hemoglobin A1c (HbA1c hemoglobin), plasma IGF-1, insulin and antidiabetic agent requirements, and homeostasis model assessment (HOMA).

Tolerability of chemotherapy will be assessed in terms of toxicities or treatment delay. Toxicities will be graded using the Common Terminology Criteria for Adverse Events (CTCAE) v 4.0.

Effects on PFS and OS will be analyzed. PFS is defined as the time elapsed from treatment initiation to first tumor progression (according to the RECIST v1.1 criteria), or death from any cause; censored are patients alive or lost to follow-up [45, 56]. OS is defined as the time elapsed from treatment initiation to death from any cause, or last follow-up.

Exercise tolerance will be monitored using the booklet, visual scales (dyspnea, muscular pain), and a Borg scale. The effects of physical activity on deconditioning, cardiorespiratory function, and muscle strength will be evaluated using the International Physical Activity Questionnaire (IPAQ), the resting heart rate and blood pressure, a 6-minute walking test with monitoring of the heart rate during exertion, a maximal strength test with elastic bands, and bioimpedancemetry. Adherence to the APA program will be registered in the patient booklet and during video calls.

Data will be collected for the 24-month follow-up period after enrollment. Tables 1 and 2 provide a summary of the study activities, measures, and schedule of visits.

**Table 1:** Schedule of visits and measures in the control arm

	Screening visit	Randomization visit	Study visit							End of treatment	Follow-up visit										
	D7	D0	W2	W4	W6	W8	W10	W12	W14	W16	W20	M6	M8	M10	M12	M14	M16	M18	M20	M22	M24
Informed consent	X																				
Randomization		X																			
Inclusion criteria	X																				
Physical activity partner	X																				
Physical activity level GPAQ	X																				
Demographic and clinical data	X																				
Concomitant treatment	X	X	X	X	X	X	X	X	X	X	X	X	X	X	X	X	X	X	X	X	X
Pathological data	X																				
Clinician visit	X	X				X				X		X	X	X	X	X	X	X	X	X	X
Physical exam	X	X	X	X	X	X	X	X	X	X	X	X	X	X	X	X	X	X	X	X	X
ECG	X																				
Questionnaires		X				X				X	X	X	X	X	X	X	X	X	X	X	X
6-minute walking test		X				X				X		X			X			X			X
Maximal strength test		X				X				X		X			X			X			X
Body composition		X				X				X		X			X			X			X
Physical activity level IPAQ		X				X				X		X			X			X			X
Biology tests																					
*Type 1*		X				X				X		X			X			X			X
*Type 2*	X		X		X		X		X		X		X	X		X	X		X	X	
*Type 3*				X				X													
*Biobank (optional)*		X																			
TAP-CT	X					X^a^				X		X	X	X	X	X	X	X	X	X	X
CA-19.9	X					X^a^				X		X	X	X	X	X	X	X	X	X	X
Adverse events			X	X	X	X	X	X	X	X	X	X	X	X	X	X	X	X	X	X	X

*CA 19–9* carbohydrate antigen 19–9, *CRP* C-reactive protein, *GPAQ* Global Physical Activity Questionnaire, *HbA1c* glycated hemoglobin, *HOMA* homeostasis model assessment, *IGF-1* insulin-like growth factor 1, *IPAQ* International Physical Activity Questionnaire, *TAP-CT* thoraco-abdominopelvic computed tomography

^a^A +/− 2-week time interval is considered as acceptable

*Biology tests - type 1*: blood cell count, CRP, fasting glucose, HbA1c, insulin/HOMA, IGF-1, albumin, prealbumin

*Biology tests - type 2*: blood cell count, CRP, fasting glucose, albumin, prealbumin

*Biology tests - type 3*: blood cell count, CRP, fasting glucose, insulin/HOMA, IGF-1, albumin, prealbumin

X: to be performed at this timepoint

**Table 2:** Schedule of visits and measures in the intervention arm

	Screening visit	Randomization visit	Study visit							End of treatment	Follow-up visit										
	D-7	D0	W2	W4	W6	W8	W10	W12	W14	W16	W20	M6	M8	M10	M12	M14	M16	M18	M20	M22	M24
Informed consent	X																				
Randomization		X																			
Inclusion criteria	X																				
Physical activity partner	X																				
Physical activity level GPAQ	X																				
Demographic and clinical data	X																				
Concomitant treatment	X	X	X	X	X	X	X	X	X	X	X	X	X	X	X	X	X	X	X	X	X
Pathological data	X																				
Clinician visit	X	X		X		X				X		X	X	X	X	X	X	X	X	X	X
APA professional		X								X		(X)			(X)			(X)			(X)
Dietitian		X	X	X		X		X		X		(X)			(X)			(X)			(X)
Physical exam	X	X	X	X	X	X	X	X	X	X	X	X	X	X	X	X	X	X	X	X	X
ECG	X																				
Questionnaires		X				X				X	X	X	X	X	X	X	X	X	X	X	X
6-minute walking test		X				X				X		X			X			X			X
Maximal strength test		X				X				X		X			X			X			X
Body composition		X				X				X		X			X			X			X
Physical activity level IPAQ		X				X				X		X			X			X			X
Biology tests																					
*Type 1*		X				X				X		X			X			X			X
*Type 2*	X		X		X		X		X		X		X	X		X	X		X	X	
*Type 3*				X				X													
*Biobank (optional)*		X																			
TAP-CT	X					X^a^				X		X	X	X	X	X	X	X	X	X	X
CA-19.9	X					X^a^				X		X	X	X	X	X	X	X	X	X	X
Adverse events			X	X	X	X	X	X	X	X	X	X	X	X	X	X	X	X	X	X	X

*CA 19–9* carbohydrate antigen 19–9, *CRP* C-reactive protein, *GPAQ* Global Physical Activity Questionnaire, *HbA1c* glycated hemoglobin, *HOMA* homeostasis model assessment, *IGF-1* insulin-like growth factor 1, *IPAQ* International Physical Activity Questionnaire, *TAP-CT* thoraco-abdominopelvic computed tomography

^a^A 2-week time (+/−) interval is considered as acceptable

(X): only for patients pursuing the APA program after week 16

*Biology tests - type 1*: blood cell count, CRP, fasting glucose, HbA1c, insulin/HOMA, IGF-1, albumin, prealbumin

*Biology tests - type 2*: blood cell count, CRP, fasting glucose, albumin, prealbumin

*Biology tests - type 3*: blood cell count, CRP, fasting glucose, insulin/HOMA, IGF-1, albumin, prealbumin

### Sample size

To detect a difference between the two groups at a global type I error of 5 %: 1) a change of five units (standard deviation [SD] = 10) for at least one of the EORTC-QLQ-C30 targeted dimensions (global QoL, fatigue, physical functioning, and pain) with an alpha error risk of 1 % in a bilateral design (with a Bonferroni adjustment of 4 %/4 = 1 %) and a statistical power of 80 % with a mean comparison test, a total of 190 patients are required [54]; 2) or a change of two units (SD = 4) for the targeted dimension of the MFI-20 scale (physical fatigue), with an alpha error risk of 1 % in a bilateral design (with a Bonferroni adjustment of 1 %) and a power of 80 % with a mean comparison test, a total of 190 patients are required [52].

The effects of exercise on fatigue and HRQoL will be tested after 16 weeks, 6 months, and 24 months post-enrollment. An interim analysis will be performed after half of the patients have achieved at least 6 months of follow-up using the O’Brien-Fleming method (to reject the null hypothesis [H0; efficacy] or to reject the study hypothesis [H1; futility]). All analysis will be performed using EAST software v5.

An arm will be considered superior to the other arm if at least one of the targeted dimensions of the EORTC-QLQ-C30 is significantly superior without any targeted dimension being superior in the other arm. Otherwise, at least three out of the five targeted dimensions will be required to be significantly superior in order to consider that one of the study arms is globally superior to another arm.

To anticipate a dropout of 5 % (that is, discontinuation of participation and loss to follow-up), a total of 200 patients will be randomized. In order to show that median TUDD for one of the targeted dimensions of the EORTC-QLQ-C30 shows change from 5 months to 8.5 months (HR = 0.58) with an alpha error risk of 1 % in a bilateral design (with a Bonferroni adjustment of 5 %/5 = 1 %) and a power of 80 %, a total of 200 patients with a minimal 12-month follow-up will be required to observe 170 events [20].

### Data analysis

Descriptive statistics will be used to characterize the study population and the instrument scores at baseline and at follow-up. Statistical analyses will be performed with SAS v9.2, Stata v12, or R v2.15. All primary statistical analyses will be conducted on the intention-to-treat population. A *P* value of < 0.01 and of < 0.05 will be considered statistically significant for the primary endpoint and secondary endpoints, respectively. Results will be presented for the total study population and according to center, cancer stage, chemotherapy schedule, ECOG PS, and baseline physical activity level.

The study data analysis will be conducted according to the following steps:

1. Description of the study sample Patient characteristics will be described for all the demographic and clinical data recorded at inclusion. Qualitative variables will be presented using absolute numbers and percentages, together with their 95 % confidence intervals (CI). Quantitative variables will be reported using mean (±SD) or median (min/max). Interaction tests will be performed. The comparison of means will be carried out using Student’s *t*-test, the Wilcoxon rank-sum test, analysis of variance (ANOVA), and the Kruskall-Wallis test, as appropriate. The association of qualitative variables will be carried out using chi-square or Fisher’s exact tests, as appropriate.

2. Score calculation and management of missing data Questionnaire scores will be calculated according to the authors’ recommendations. Patients with missing data will be excluded from analysis. If data are missing at random, the average of items answered in the dimension will be measured, and if data are not missing at random, a multiple imputation method will be used to complete the dataset.

3. Statistical analysis of the primary endpoint Absolute (mean [SD] and median [min/max]) scores for fatigue and HRQoL at inclusion and at each time point will be measured and compared between the two groups. An ANOVA will be carried out after determining the normality of the distribution of the scores. Univariate analyses of variance will be performed to compare the targeted dimensions at week 16 between the two groups, to calculate the average of the difference, and to identify potential confounders in the relationship between the exercise intervention, fatigue, and HRQoL. Factors with a statistical threshold of 5 % will be integrated into a multivariate ANOVA. The results will be presented with 99 % CI.

4. Statistical analysis of secondary endpoints TUDD scores for fatigue, targeted HRQoL dimensions, OS, and PFS will be estimated by the Kaplan-Meier method and compared using a log-rank test. The effect of the intervention will be expressed as hazard ratio (HR) with a 99 % CI. The Cox regression model will be used for multivariate analysis to measure the effect of the intervention on the five targeted dimensions. Different definitions of TUDD and types of events will be investigated in sensitivity analyses [20]. For the analysis of anxiety/depression and pain data, the variance analysis models will be used to assess whether, compared to baseline measurements, patients show changes in scores following the interventions (at each time point). Continuous data will be checked for normal distribution and, where applicable, log transformation will be applied to the data. The intraclass correlation coefficients for outcome variables of interest will be calculated. Univariate and multivariate logistic regression analyses of grade 3–4 toxicities and 3–4 late complications will be performed. Weight and BMI variables, as well as physical activity level and nutritional status variables, will be compared between groups. The HADS questionnaire will be completed by the physical activity partner and analyzed for descriptive purposes.

## Discussion

Fatigue and altered HRQoL are common symptoms in patients with cancer receiving chemotherapy. Adapted physical activity during treatment is an original and promising non-pharmaceutical strategy that may help better manage these symptoms in supportive and palliative care interventions. Previous data have indicated that APA is feasible and efficient in various cancers. Effects of an exercise intervention in patients with advanced PDAC have never been explored. As these patients are strongly affected by fatigue, we hypothesized that they are likely to benefit from the exercise intervention. In addition, exercise has been reported to have a beneficial effect on tumor outcomes by reducing insulin resistance and insulin and IGF-1 secretion.

In this study, characteristics of patients with advanced PDAC that may affect the implementation of the APA program were taken into account. We propose an original method: the introduction of the physical activity partner to optimize patient adherence. This approach is based on the hypothesis that patient group sessions may be psychologically counterproductive in advanced PDAC patients because of the likely risk of short-term degradation of PS in several participants. Moreover, given that nutrition is a critical issue in the management of patients with PDAC and that physical exercise may increase patient denutrition by increasing energy expenditures, we carefully adapted a nutritional intervention to the APA program.

In conclusion, the APACaP study is a large multicenter randomized controlled trial to assess the feasibility and the efficacy of an unsupervised 16-week APA program on fatigue and HRQoL in patients with advanced PDAC. This intervention may appear challenging in those patients, given multiple cancer-related symptoms (fatigue, depression, pain, and denutrition). However, we hypothesize that the APA program may be beneficial for patients with advanced PDAC and may improve PDAC-related symptoms and HRQoL. If the APA program proves to be feasible and effective in this population, systematic implementation of this approach in association with anti-tumoral treatment will be the next logical step in this setting. Finally, the study results may also have implications for the multimodal treatment of other digestive cancers.

## Trial status

APACaP started enrolling patients in September 2014. Study is ongoing, and the completion date is estimated at December 2017.

## Additional file

Additional file 1: Table S1. Eastern Cooperative Oncology Group (ECOG) Scale of Performance Status. (DOC 29 kb)

## Abbreviations

1-RM: One-repetition maximum
ACS: American Cancer Society
ACSM: American College of Sports Medicine
AE: Adverse event
AFSOS: Association Francophone pour les Soins Oncologiques de Support
ANSM: Agence Nationale de Sécurité du Médicament et des produits de santé
APA: Adapted physical activity
CA 19–9: carbohydrate antigen 19–9
CRP: C-reactive protein
CTCAE: Common Terminology Criteria for Adverse Events
ECOG: Eastern Cooperative Oncology Group
EORTC: European Organization for Research and Treatment of Cancer
FDR: Fixed-dose rate
FOLFIRINOX: 5-fluorouracil plus irinotecan plus oxaliplatin combination
GPAQ: Global Physical Activity Questionnaire
HADS: Hospital Anxiety and Depression Scale
HbA1c: glycated hemoglobin
HOMA: Homeostasis model assessment
HR: Hazard ratio
HRQoL: Health-related quality of life
IGF-1: Insulin-like growth factor 1
INCa: Institut National du Cancer
IPAQ: International Physical Activity Questionnaire
MFI-20: Multidimensional Fatigue Inventory
OS: Overall survival
PDAC: Pancreatic ductal adenocarcinoma
PFS: Progression-free survival
PS: Performance status
RECIST: Response evaluation criteria in solid tumors
RR: Relative risk
SD: Standard deviation
SFNEP: Société Francophone de Nutrition Clinique et Métabolisme
SHBG: Sex hormone binding globulin
TAP-CT: Thoraco-abdominopelvic computed tomography
TUDD: Time until definitive deterioration
WHO: World Health Organization
