# Supplementary material for: Rationale and design of the Adapted Physical Activity in advanced Pancreatic Cancer patients (APACaP) GERCOR (Groupe Coopérateur Multidisciplinaire en Oncologie) trial: study protocol for a randomized controlled trial
Source: Trials. 2015 Oct 12;16:454. doi: 10.1186/s13063-015-0983-8 (PMC4603729; doi:10.1186/s13063-015-0983-8)
Supplement: Additional file 1: Table S1. — Eastern Cooperative Oncology Group (ECOG) Scale of Performance Status. (DOC 29 kb) [file 13063_2015_983_MOESM1_ESM.doc]

Additional file 1: Table S1. Eastern Cooperative Oncology Group Performance Status Scale.

| **Grade** | **Definition** |
| --- | --- |
| **0** | Fully active, able to carry on all pre-disease performance without restriction |
| **1** | Restricted in physically strenuous activity but ambulatory and able to carry out work of a light or sedentary nature, e.g., light house work, office work |
| **2** | Ambulatory and capable of all self-care but unable to carry out any work activities; up and about more than 50% of waking hours |
| **3** | Capable of only limited self-care; confined to bed or chair more than 50% of waking hours |
| **4** | Completely disabled; cannot carry on any self-care; totally confined to bed or chair |
| **5** | Dead |

ECOG: Eastern Cooperative Oncology Group (ECOG
